# Research Progress on the Antiviral Activity of Glycyrrhizin and its Derivatives in Liquorice

**DOI:** 10.3389/fphar.2021.680674

**Published:** 2021-07-06

**Authors:** Changchao Huan, Yao Xu, Wei Zhang, Tingting Guo, Haochun Pan, Song Gao

**Affiliations:** ^1^ Institutes of Agricultural Science and Technology Development, College of Veterinary Medicine, Yangzhou University, Yangzhou, China; ^2^ Jiangsu Co-Innovation Center for Prevention and Control of Important Animal Infectious Diseases and Zoonoses, Yangzhou, China; ^3^ Key Laboratory of Avian Bioproduct Development, Ministry of Agriculture and Rural Affairs, Yangzhou, China; ^4^ College of Medicine, Yangzhou University, Yangzhou, China

**Keywords:** liquorice, triterpenoid, antiviral activity, glycyrrhizin, glycyrrhetinic acid

## Abstract

Liquorice is a traditional medicine. Triterpenoids such as glycyrrhizin and glycyrrhetinic acid are the main active constituents of liquorice. Studies have revealed that these compounds exert inhibitory effects on several viruses, including SARS-CoV-2. The main mechanisms of action of these compounds include inhibition of virus replication, direct inactivation of viruses, inhibition of inflammation mediated by HMGB1/TLR4, inhibition of β-chemokines, reduction in the binding of HMGB1 to DNA to weaken the activity of viruses, and inhibition of reactive oxygen species formation. We herein review the research progress on the antiviral effects of glycyrrhizin and its derivatives. In addition, we emphasise the significance of exploring unknown antiviral mechanisms, structural modifications, and drug combinations in future studies.

## Introduction

Liquorice has been documented as a herbal medicine in ancient medical books of China, India, and Greece and has been used for thousands of years (Zeng et al., 1988). Primarily, it is used in clinics to treat diseases of the respiratory, digestive, and immune systems (Deng et al., 2017; Sun et al., 2018; Yan et al., 2018; Yao and Sun, 2019; Paudel et al., 2020; Yang et al., 2020). Liquorice has favourable preventive and therapeutic effects on various diseases, with fewer side effects (Kwon et al., 2020); therefore, research and mining of potential pharmacological activities of liquorice have been a hot topic in the field of pharmacology. Currently, many experimental results have shown that liquorice and its extract have antibacterial, antiviral, anti-inflammatory, anticancer, antioxidant, liver protection, neuroprotection, skin whitening, hypoglycaemic, memory-enhancing, and other biological activities (Petramfar et al., 2020), indicating that liquorice holds a great developmental and application prospect in cosmetic production and in the treatment of liver diseases, diabetes, ischaemia–reperfusion injury, Alzheimer’s disease, Parkinson’s disease, epilepsy, depression, and cancer (Pastorino et al., 2018; Li et al., 2019; Zhang et al., 2021).

**FIGURE 1:**
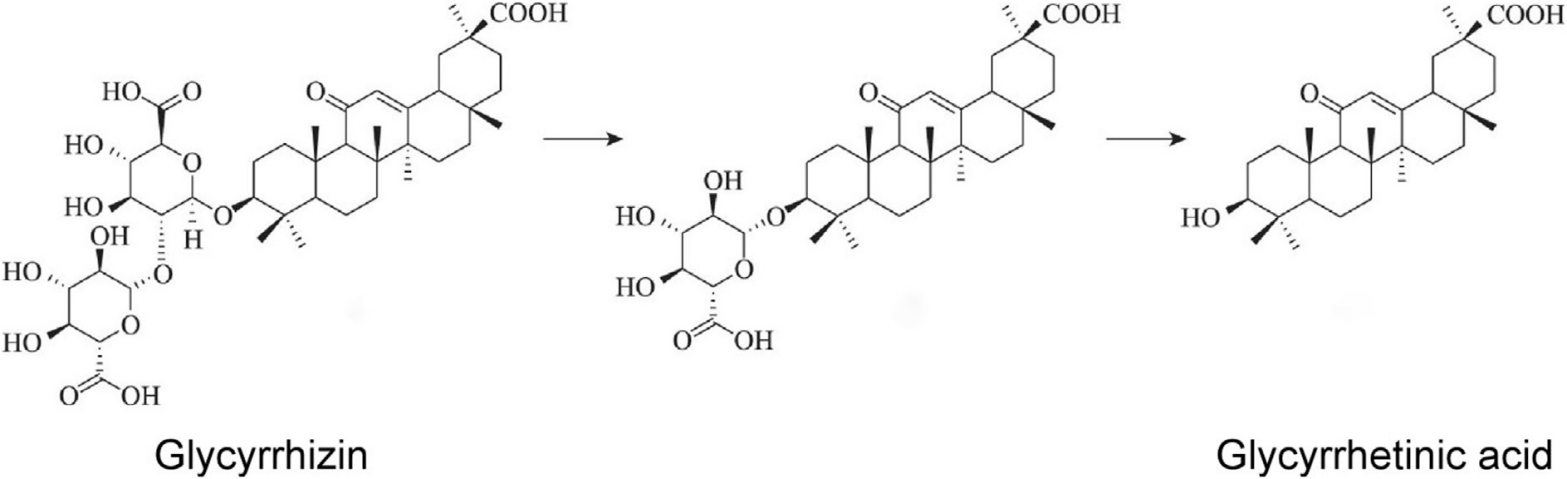
The structure and metabolic relationship between glycyrrhizin and glycyrrhetinic acid.

Glycyrrhizin (also known as glycyrrhizic acid or glycyrrhizinic acid) (Drugs B.I., 2006) and glycyrrhetinic acid, which are the most important chemical constituents of liquorice, belong to the class triterpenoids. Among all saponins in liquorice, glycyrrhizin is present in the highest amount (>2%), and its content in wild, high-quality liquorice can be as high as 7% (Zhang and Ye, 2009). Glycyrrhizin is metabolised through the gastrointestinal tract to produce glycyrrhetinic acid (Figure 1) (Yang, 2020), which also possesses many pharmacological properties, and its most interesting property is its antiviral activity. Glycyrrhizin exerts inhibitory effects on hepatitis C virus (HCV) (Zhang et al., 2020), herpes simplex type 1 (HSV-1) (Hirabayashi et al., 1991), influenza virus (Wolkerstorfer et al., 2009), and severe acute respiratory syndrome (SARS)-associated coronaviruses (Cinatl et al., 2003). This article reviews the research progress on the antiviral activity of glycyrrhizin and its derivatives in liquorice to provide a reference for further scientific research and for guiding clinical applications. Although only a few reports are available on the role of glycyrrhizin in herpes virus and human immunodeficiency virus (HIV) infections, we present information about herpes virus and HIV based on studies by Pu et al. (2013) and Huan et al. (2020), and these aspects form the basis of the present review. Overall, our review discusses advances in research on the antiviral properties and mechanism of action of glycyrrhizin.

## Anti-Herpes Virus Activity

Herpes virus is one of the most common viruses infecting animals and humans. More than 90% adults have been infected with at least one or more types of herpes viruses, and the viruses usually establish a latent infection (Chayavichitsilp et al., 2009). Some herpes viruses lead to lifelong infections, which are common worldwide (Bradley et al., 2014; Okonko and Cookey, 2015; Reward et al., 2019).

In 1979, a study reported that glycyrrhizic acid could directly inactivate HSV [multiplicities of infection (MOI) = 5], and this effect was found to be irreversible (Pompei et al., 1979). Subsequently, some researchers detected the anti-HSV effects of glycyrrhetinic acid derivatives, sodium carbenoxolone sodium (CBX), and cicloxolone sodium (CCX) (Dargan and Subak-Sharpe, 1986). Through *in vivo* and *in vitro* studies, researchers found that 500 μM CBX and 300 μM CCX have anti-HSV-1 and anti-HSV-2 (MOI = 5) activities, and thus can significantly inhibit virus replication and reduce the number of infectious virus particles by 10,000–100,000 times. Glycyrrhizin inhibits HSV-1 replication *in vitro,* and its IC_50_ is 0.5 mM (Hirabayashi et al., 1991). Sekizawa et al. (2001) revealed that glycyrrhizin could increase the survival rate of HSV-1-infected mice. Ikeda et al. (2005) found that glycyrrhizin and glycyrrhetic acid inhibited HSV-1 replication, and the effect of glycyrrhetic acid was 10 times greater than that of glycyrrhizin. Glycyrrhizin was also shown to reduce adhesion between cerebral capillary vessel endothelial cells and polymorphonuclear leukocytes in the treatment of HSV infection (Huang et al., 2012).

Curreli et al. (2005) found that latent infection with kaposi sarcoma-associated herpesvirus (KSHV) in B lymphocytes could be terminated using glycyrrhizic acid. Glycyrrhizic acid was found to block the latent KSHV infection by upregulating the expression of viral cyclin (ORF72) and downregulating the expression of latency-associated nuclear antigen (LANA), thereby selectively inducing death of KSHV-infected cells. Glycyrrhizic acid was also reported to inhibit KSHV replication in patients with hepatitits B virus (HBV) infection (Xie et al., 2011). In addition, Kang and Lieberman (2011) found that glycyrrhizic acid decreases transcription during KSHV latency to inhibit virus replication. They confirmed that glycyrrhizic acid disrupts the RNA polymerase II (RNAPII) complex and alters the enrichment of the RNAPII-pausing complex. Furthermore, glycyrrhizic acid was shown to reduce the interaction between SMC3 and RAD21 and between SPT5 and RNAPII. Moreover, it abrogatated RNAPII to pause at intragenic CTCF-cohesion binding sites, leading to reduced mRNA production and a defect in sister chromatid cohesion.

Lin (2003) proved that glycyrrhizic acid dose-dependently inhibits the Epstein–Barr virus (EBV) replication. Glycyrrhizic acid mainly plays an antiviral role in the early stage of the EBV replication cycle; however, it does not affect the adsorption and inactivation of virus particles. Lin et al. (2008) found that seven derivatives of glycyrrhizic acid could inhibit EBV infection in a dose-dependent manner, and the antiviral activity of these derivatives was enhanced when amino acid residues were introduced into the carbohydrate part of glycyrrhizic acid. The authors also reported that 18β-glycyrrhetinic acid (the metabolic product of glycyrrhizic acid) is 7.5-fold more active against EBV than glycyrrhizic acid (Lin et al., 2008).

Cellular SUMOylation processes are the proposed targets for antiviral therapies. Bentz et al. (2019) found that glycyrrhizic acid inhibits SUMOylation processes, limits cell growth, and induces apoptosis in multiple cell lines. Glycyrrhizic acid acts on the first step of the SUMOylation process and leads to low levels of spontaneous EBV reactivation. Glycyrrhizic acid was not found to affect the induced reactivation of the virus; however, its extract was shown to reduce the ability of the virus to infect the remaining cells (Lin et al., 2008). Therefore, glycyrrhizic acid may be beneficial in the treatment of EBV-related malignant tumours and other diseases caused by the SUMOylation process.

## Anti-Hepatitis Virus Activity

Glycyrrhizic acid exhibits direct anti-hepatitis virus activity and low toxicity in host cells (Romero et al., 2005). It has been used to treat chronic hepatitis for many years in Japan. Glycyrrhizin can inhibit the secretion of HBV surface antigen (HBsAg) in PLC/PRF/5 cells *in vitro*, which leads to its accumulation in the cytoplasmic vacuoles of the Golgi apparatus, changes in the intracellular transport of HBsAg, and inhibition of its sialylation in a dose-dependent manner (Takahara et al., 1994). Sato et al. (1996) revealed that glycyrrhizin could bind to hepatocytes at a certain concentration, change the expression of HBV-related antigens on hepatocytes, and inhibit the sialylation of HBsAg. Matsuo et al. (2001) revealed that glycyrrhizin in combination with lamivudine may inhibit HBV replication in an HBV carrier with non-Hodgkin lymphoma.

Glycyrrhizin can not only effectively inhibit HBV infection but also exhibit a strong anti-HCV activity. Ashfaq et al. (2011) used a nontoxic dose of glycyrrhizin to detect its antiviral activity in HCV-infected hepatocytes. The study results revealed that glycyrrhizin can decrease the titre of hepatitis C virus (2 × 10^5^ copies of HCV infection) by 50% at a concentration of 7 ± 1 μg/ml. Additionally, its inhibitory effect increased when it was used in combination with interferon. In addition to interferon (Fujisawa, 1991), glycyrrhizin is used in combination with various drugs to treat hepatitis virus infection, and its use in combination with other drugs has yielded superior clinical outcomes (Tandon et al., 2001; Tarao, 2001; Hidaka et al., 2007; Chen et al., 2017). A glycyrrhizin-containing preparation, Stronger Neo-Minophagen C^TM^ (SNMC), protects mitochondria by reducing oxidative stress during HCV treatment in transgenic mice (Korenaga et al., 2011). Matsumoto et al. (2013) demonstrated that glycyrrhizin can decrease the release of infectious HCV particles by inhibiting PLA2G1B.

A recent study reported that glycyrrhetinic acid could inhibit the release of high-mobility group box 1 (HMGB1), block the cytokine activity of HMGB1, and significantly improve the liver inflammatory injury induced by mouse hepatitis virus (1 × 10^4^ PFU/mouse) through the HMGB1-TLR4 signalling pathway. This protective effect is related to a substantial reduction in IL-17 and IL-22 levels rather than the direct inhibition of intracellular virus replication (Shi et al., 2020).

## Anti-Influenza Virus Activity

The effect of glycyrrhizic acid on influenza virus was reported in the early 1980s. *In vitro* experiments by Pompei et al. (1983) demonstrated that glycyrrhizic acid could inhibit the replication of influenza virus in chicken embryos by reducing the haemagglutinin level. The mechanism of inhibition of influenza virus replication by glycyrrhizic acid has been reported (Wolkerstorfer et al., 2009; Michaelis et al., 2010; Sakai-Sugino et al., 2017). A combination of HMGB1 with influenza virus nucleoprotein can promote the growth of influenza virus and enhance the activity of virus polymerase (Moisy et al., 2012). Glycyrrhizin can reduce the activity of influenza virus polymerase by antagonising this binding effect, thus inhibiting influenza virus replication (Paudel et al., 2020). Michaelis et al. (2011) found that 200 μg/ml glycyrrhizin could significantly reduce the cytopathic effect (CPE) caused by H5N1 influenza virus infection at an MOI of 0.01, 0.1, or 1 in A549 cells. The therapeutic concentration of glycyrrhizin interferes with the replication of highly pathogenic H5N1 influenza A virus at least partly by interfering with the formation of reactive oxygen species (ROS) induced by H5N1, thereby reducing the activation of p38, JNK, and NF-kB in lung cells. Glycyrrhizin was also found to inhibit H5N1-induced production of CXCL10, IL-6, and CCL5 (MOI = 2) and H5N1-induced apoptosis without affecting virus replication and NK cell activity (Michaelis et al., 2010).

The combination of glycyrrhizic acid with various drugs produces a synergistic effect, which protects against influenza virus infection. Smirnov et al. (2012) studied the regulatory effects of glutamyl-tryptophan (EW) and glycyrrhizic acid and their combination on influenza A (H3N2) virus infection in mice (1 or 10 LD_50_). The results revealed that compared with EW alone, glycyrrhizic acid (10 mg/kg body weight) in combination with EW (0.1 μg/kg, 10 μg/kg, and 1,000 μg/kg) produced significant antiviral effects, which alleviated lung oedema and inflammatory cell infiltration. Use of glycyrrhizic acid in combination with ribavirin, a broad-spectrum antiviral drug, significantly inhibited lung consolidation in mice infected with H1N1 influenza virus. A combination of 50 mg kg−^1^ d−^1^ glycyrrhizin and 40 mg kg−^1^ d−^1^ ribavirin was found to provide 100% protection to infected mice (5 times LD50 of influenza H1N1 virus infection), suggesting that the combination of ribavirin and glycyrrhizin has a potential clinical value (Chen et al., 2015b).

## Anti-Human Immunodeficiency Virus Activity

Studies have shown that glycyrrhizin inhibits HIV replication in a dose-dependent manner (Ito et al., 1987; Ito et al., 1988; Hattori et al., 1989). Ito et al. (1988) found that glycyrrhizin at a concentration of 0.6 mM completely inhibited HIV-induced (MOI = 0.002) MT-4 cell plaque formation as well as HIV-induced cytopathogenicity in MT-4 and MOLT-4 cells *in vitro* (Hirabayashi et al., 1991).

In addition, glycyrrhizin increases the number of OKT4 lymphocytes and improves liver dysfunction and thus prevents the progression of haemophilia to AIDS in HIV-positive patients with haemophilia (Mori et al., 1989). Glycyrrhizin can not only inhibit HIV replication but also affect the entry of HIV into cells. HIV requires chemokine receptors or chemokine receptor-like molecules to enter cells (Cocchi et al., 1996; Feng et al., 2011). Sasaki et al. (2002) found that glycyrrhizin could induce the production of CC chemokine ligand (CCL) 4 and CCL5 in cultures of peripheral blood mononuclear cells from HIV-infected patients. These β-chemokines can compete with HIV to bind with chemokine receptors, thus inhibiting the entry of NSI-HIV. CC chemokine receptor 5 (CCR5) or CXC-chemokine receptor 4 (CXCR4) are the coreceptors necessary for HIV-1 entry into cells (Alkhatib et al., 1996; Dragic et al., 1996). Yoshida et al. (2009) and Takei et al. (2005) confirmed that glycyrrhizin can greatly inhibit the production of CCL2 and interleukin 10 (IL-10). Consequently, CCR5 expression, which is mediated by CCL2 or IL-10, reduces significantly, resulting in effective inhibition of the entry of HIV into cells.

Glycyrrhizin can also reduce the fluidity of the cell membrane, which reduces intercellular fusion, thus inhibiting the spread of HIV across cells. Harada (2005) washed the cells treated with glycyrrhizin, added the replacement medium lacking glycyrrhizin, and observed a time-dependent increase in the fluidity of the membrane as well as in the sensitivity of the cells to infection and fusion. This finding provides a novel strategy for the prevention and treatment of enveloped viruses.

## Anti- SARS-CoV Activity

Researchers began investigating the role of glycyrrhizin in protection against SARS-associated coronavirus (SARS-CoV) infection after the outbreak of SARS in 2003. Cinatl et al. (2003) evaluated the antiviral effects of five drugs, including ribavirin and glycyrrhizin, on SARS-CoV, and glycyrrhizin was found to exhibit the strongest inhibitory effect on SARS-CoV replication in Vero cells. Furthermore, a study found that glycyrrhizin can inhibit the early stages of the virus replication cycle, namely adsorption and penetration, and the effect of glycyrrhizin addition on virus adsorption was not found to be as good as that of glycyrrhizin addition after virus adsorption. However, Chen et al. (2004) could not detect the inhibitory effect of glycyrrhizin on SARS-CoV in the fRhK-4 cell line. The modification of the glycyrrhizin structure, particularly the production of amide derivatives and amino acid conjugates, can significantly improve its anti-SARS-CoV activity, although this modification increases its cytotoxicity (Hoever et al., 2005). In clinical trials, clinical symptoms such as dyspnoea improved rapidly (Lu et al., 2003), the average time for lung lesion improvement from the most severe to 50% decreased, and no side effects were observed in the glycyrrhizin treatment group (Wu et al., 2004).

SARS-CoV-2 is a novel coronavirus, and the coronavirus disease 2019 pandemic caused by SARS-CoV-2 was named coronavirus disease-2019 (COVID-2019) by the World Health Organisation (WHO) (Coronaviridae Study Group of the International Committee on Taxonomy of, 2020; Li et al., 2020; Zhu et al., 2020). SARS-CoV-2 and SARS-CoV gene sequences share 79.5% homology (Zhou et al., 2020), and many similarities have been observed in the clinical symptoms of the infections caused by these two viruses (Wang et al., 2020). Therefore, whether glycyrrhizin shows antiviral activity against SARS-CoV-2 similar to SARS-CoV remains to be investigated (Bailly and Vergoten, 2020). Luo et al. (2020) explored the potential pharmacological effects of glycyrrhizin in COVID-19 treatment. The authors found that glycyrrhizin exerts various pharmacological effects such as angiotensin-converting enzyme II (ACE2) binding, proinflammatory cytokine downregulation, endogenous interferon induction, inhibition of intracellular R accumulation and thrombin, and excessive production of airway exudates. These findings suggest that glycyrrhizin may be a promising drug for COVID-19 treatment (Ding et al., 2020; Murck, 2020; Chrzanowski et al., 2021).

In a recent study, glycyrrhizin significantly inhibited SARS-CoV-2 (MOI = 0.01) replication in Vero E6 cells in a dose-dependent manner and exhibited no significant cytotoxicity (Gowda et al., 2021). Glycyrrhizin can also prevent virus replication (100 TCID_50_ of SARS-CoV-2) by inhibiting the viral main protease M^pro^ (Sinha et al., 2021; van de Sand et al., 2021). Using computer-aided drug design and biological verification, Yu et al. (2021) found that glycyrrhizin is the most effective and nontoxic broad-spectrum anti-coronavirus molecule *in vitro*, particularly against SARS-CoV-2.

## Effects on Some Animal Viruses

Glycyrrhizin has demonstrated antiviral activity against some animal viruses. Li et al. (2009) studied the effect of glycyrrhizin diammonium on infectious bronchitis virus (IBV) cell infection (MOI = 0.001) through CPE observation, plaque reduction test, and RT-PCR. The results revealed that glycyrrhizin diammonium has a direct antiviral activity and that it can completely inhibit cell infection. Glycyrrhizin alone or in combination with duck hepatitis virus (DHV) vaccine has demonstrated good immune stimulant and antiviral effects against DHV (Soufy et al., 2012; Okda et al., 2013). Dipotassium glycyrrhizinate can directly inactivate and/or interfere with infectious bursal disease virus (IBDV) replication and effectively inhibit IBDV infection (100 TCID_50_, 1 × 10^−1.5^) *in vitro* (Sun et al., 2013). Li et al. (2014) found that diammonium glycyrrhizinate (DG) exerts an antiviral effect on porcine testicular (ST) cells infected with PPV (100 TCID_50_), and DG was found to have a strong inhibitory effect on PPV when the virus was treated before incubation. DG also demonstrated its antiviral activity against Marc-145 cells infected with porcine reproductive and respiratory syndrome virus (PRRSV). PRRSV could be effectively controlled through inhibition of viral replication and N gene expression and through reduction of apoptosis (Wang et al., 2013). Studies have also reported that glycyrrhizin mainly inhibits the penetration of PRRSV and has a slight effect on the adsorption or release of PRRSV during its life cycle (Duan et al., 2015). By using carbon dots (CDs) with high biocompatibility and glycyrrhizic acid, Tong et al. (2020) synthesised Gly-CDs through the hydrothermal method. These Gly-CDs could inhibit the invasion and replication of PRRSV, stimulate the innate immune response of antivirus, and inhibit the accumulation of intracellular ROS caused by PRRSV infection (MOI = 1). Glycyrrhizin can also inhibit the entry and replication of porcine epidemic diarrhoea virus (PEDV) but does not affect the assembly and release of the virus (Huan et al., 2017). Inhibition of PEDV infection (MOI = 0.1) and secretion of proinflammatory cytokines occurred mainly through the HMGB1/TLR4-MAPK p38 pathway (Gao et al., 2020).

## Discussion

**FIGURE 2:**
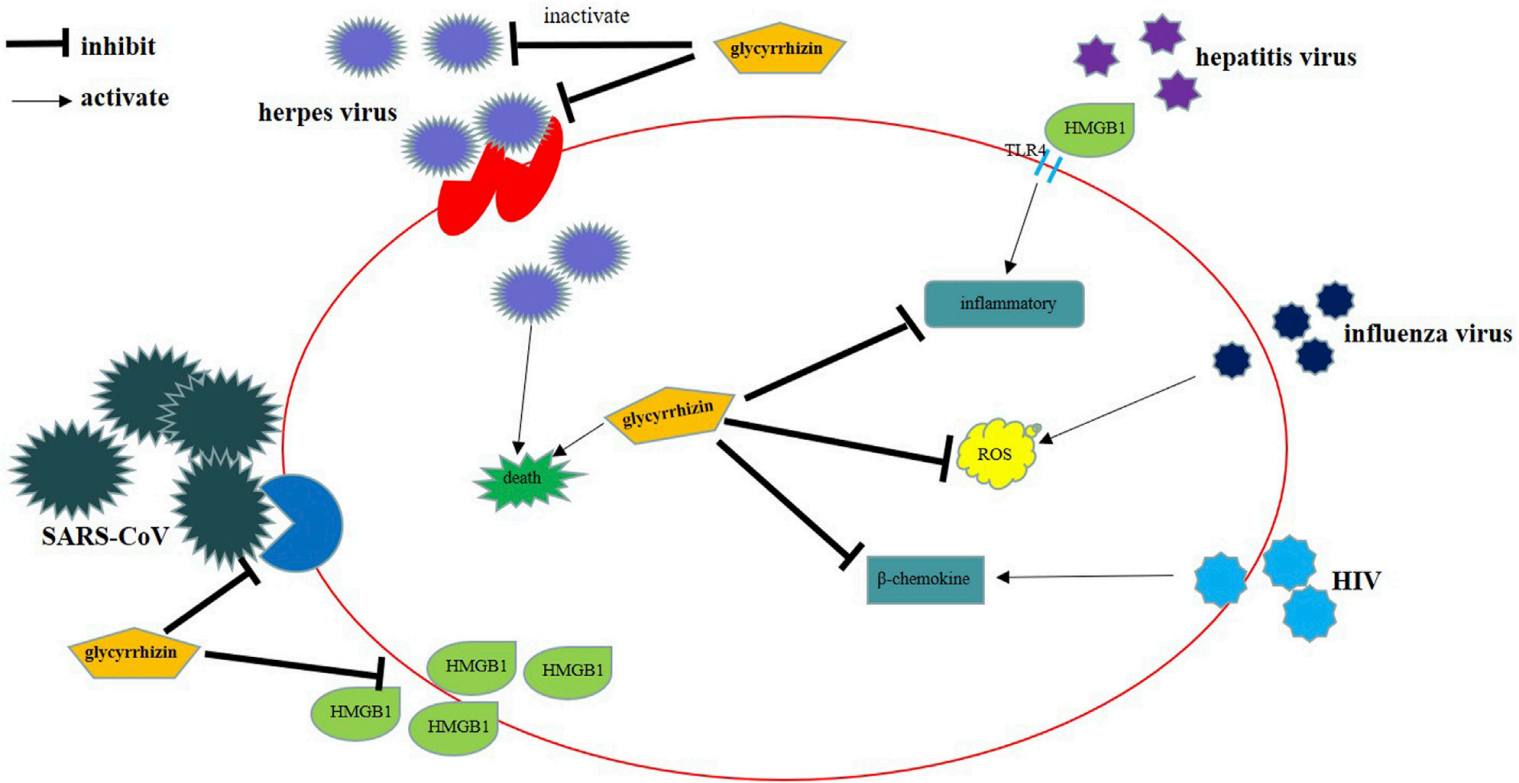
The mechanisms of action of glycyrrhizin against viruses.

Glycyrrhizin and its derivative protect against many viruses through their antiviral activity (Table 1). The main mechanisms of action of these compounds include inhibition of virus replication, direct inactivation of viruses, inhibition of the replication and expression of viral genes, increase in cell death, inhibition of inflammation mediated by HMGB1/TLR4, inhibition of β-chemokines, reduction in HMGB1 release, reduction in the fluidity of the cell membrane, reduction in the binding of HMGB1 to DNA to weaken the activity of viruses, and inhibition of the formation of ROS (Figure 2). The mechanisms of action of glycyrrhizin against viruses are complex; however, many mechanisms remain unknown. Therefore, elucidation of the antiviral mechanism of glycyrrhizin may help in developing strategies to enhance its antiviral activity and is a subject worthy of further studies. In our further studies, we aim to focus on exploring the mechanisms through which glycyrrhizin induces direct inactivation of viruses and inflammatory and immune responses.

**TABLE 1:** The antiviral activity and mechanism of actions of glycyrrhizin and its derivatives.

Virus		Mechanisms of action	Glycyrrhizin and its derivatives	References
Herpes virus	HSV-1	Directly inactivates herpes simplex virus	Glycyrrhizic acid (8 mM)	Pompei et al. (1979)
	HSV-1, HSV-2	Inhibition of virus replication	Carbenoxolone sodium (500 μM) and cicloxolone sodium (300 μM)	Dargan and Subak-Sharpe, (1986)
	KSHV	Upregulation of the expression of viral cyclin and downregulation of the expression of latency-associated nuclear antigen, selectively inducing cell death in KSHV-infected cells	Glycyrrhizic acid (3 mM, 4 mM)	Curreli et al. (2005)
	EBV	Inhibit virus invasion into host cells in the early stages of virus replication; inhibit the sumerization process, prevent cell proliferation, increase cell death, and prevent the resulting virus from infecting new cells	Glycyrrhizic acid (IC_50_ = 0.04 mM); glycyrrhizic acid (0.5, 1.0, 2.0, 3.0, 4.0 mM)	(Lin, 2003; Bentz et al., 2019)
Hepatitis virus	HBV	Inhibition of hepatitis B surface antigen secretion, sialylation, and intracellular transpo	Glycyrrhizin (0.5 mg/ml, 1 mg/ml, 2 mg/ml)	Takahara et al. (1994)
	HCV	Inhibition of HCV3a core gene expression at mRNA and protein levels	Glycyrrhizin (2.5 μg/ml, 5 μg/ml, 10 μg/ml, 20 μg/ml)	Ashfaq et al. (2011)
	Mouse hepatitis virus	Improvement of liver inflammatory injury through the high-mobility group box 1 (HMGB1)–TLR4 signal pathway	Glycyrrhetinic acid (10 μg/ml, 100 μg/ml, 1,000 μg/ml)	Shi et al. (2020)
Influenza virus	H5N1	Inhibits the formation of ROS induced by H5N1 and then decreases the activation of NFκB, JNK, and p38	Glycyrrhizin (25 μg/ml, 50 μg/ml, 100 μg/ml, 200 μg/ml)	Michaelis et al. (2011)
	Influenza virus	Antagonises the binding of HMGB1 to influenza virus nucleoprotein and reduces the activity of influenza virus polymerase, thus inhibiting the replication of influenza virus	Glycyrrhizin	Moisy et al. (2012)
	H5N1	Inhibits H5N1-induced CXCL10, IL-6 and CCL5 production, inhibits H5N1-induced apoptosis but does not interfere with H5N1 replication	Glycyrrhizin (50 μg/ml, 100 μg/ml, 200 μg/ml)	Michaelis et al. (2010)
HIV	HIV-1	Inhibition of virus replication by inhibiting protein kinase C	Glycyrrhizin (0.075, 0.15, 0.3, 0.6, 1.2, 2.4 mM)	Ito et al. (1988)
	NSI-HIV	Inhibition of NSI-HIV replication in PBMC by inducing the production of β-chemokines (CCL4 and CCL5)	Glycyrrhizin (0.1 μg/ml, 1 μg/ml, 10 μg/ml, 100 μg/ml)	Sasaki et al. (2002)
	HIV-1	Reduces the fluidity of cell membrane, resulting in a decrease in intercellular fusion, thus inhibiting the transmission of HIV between cells	Glycyrrhizin (0.06 mg/ml, 0.13 mg/ml, 0.25 mg/ml, 0.5 mg/ml, 1 mg/ml)	Harada, (2005)
SARS-CoV	SARS-CoV	Inhibits virus replication; inhibits the adsorption and infiltration of viruses	Glycyrrhizin (after virus adsorption, EC_50_ = 600 μg/ml; during and after virus adsorption, EC_50_ = 300 μg/ml; during virus adsorption, EC_50_ = 2,400 μg/ml)	Cinatl et al. (2003)
	SARS-CoV-2	Inhibition of HMGB1 release; inhibition of virus replication	Glycyrrhizin (10 mM, 250 mM, 1,000 μM)	Gowda et al. (2021)
Animal viruses	IBV	Direct antiviral effect, inhibit cell infection; decrease apoptosis of infected cells	Glycyrrhizin diammonium (0.0225, 0.09, 0.36, 1.44 mM)	Li et al. (2009)
	IBDV	Inhibition of virus replication, direct inactivation of virus, and inhibition of virus adsorption	Dipotassium glycyrrhizinate (EC_50_ = 663.2 ± 268.4 μg/ml)	Sun et al. (2013)
	PPV	Directly inactivates the virus	Diammonium glycyrrhizinate (250 μg/ml)	Li et al. (2014)
	PRRSV	Directly inactivates PRRSV, inhibits PRRSV invasion and replication, stimulates cells to produce interferon, and inhibits PRRSV infection-induced reactive oxygen species production	Glycyrrhizic-acid-based carbon dots (0.30 mg/ml)	Tong et al. (2020)
	PEDV	Inhibition of PEDV infection and secretion of proinflammatory cytokines through the HMGB1/TLR4-MAPKp38 pathway	Glycyrrhizin (0.1, 0.2, 0.4, 0.8 mM)	Gao et al. (2020)

COVID-19 has become a pandemic. Although vaccines have been approved, they have not been widely used and no specific treatment for COVID-19 is available to date (Al-Kamel and Grundmann, 2021). Glycyrrhizin exerts extensive antiviral effect and obvious inhibitory effects on SARS-CoV; therefore, glycyrrhizin may be an effective drug against COVID-19. Ding et al. provided the clinical data of a critically ill patient with COVID-19 who was cured by a combination of DG and vitamin C (Ding et al., 2020). SARS-CoV-2 has been shown to enter cells through angiotensin-converting enzyme 2 (ACE2) receptors (Letko et al., 2020; Wan et al., 2020; Xu et al., 2020). Therefore, targeting ACE2 could be a promising strategy to prevent SARS-CoV-2 infection. Glycyrrhizin was recently shown to have the potential to bind to ACE2 (Zhou and Huang, 2020). All these findings suggest that the use of glycyrrhizin could be promising in COVID-19 treatment.

Structural modification of natural products is one of the approaches to discover novel drugs. Through structural modification, the potency and solubility of natural products can be increased, pharmacokinetic properties such as absorption and distribution can be improved, and side effects can be eliminated or reduced (Chen et al., 2015a). Many artificially modified natural product derivatives, such as artemisinin (antimalaria) and paclitaxel (anticancer), have been successfully used in clinics (Yao et al., 2017). Some glycyrrhizin derivatives have demonstrated stronger antiviral activity than glycyrrhizin, although with higher cytotoxicity (Hoever et al., 2005; Baltina et al., 2019; El-Senduny et al., 2019). Therefore, structural modification of glycyrrhizin in the future could be valuable for identifying glycyrrhizin derivatives with the highest antiviral activity and least cytotoxicity. The use of a combination of two or more drugs is another approach to improve efficacy and reduce side effects of drugs. Since the approval of the first batch of combination drugs by FDA in the 1940s, combination drugs have become invaluable for clinical application (Das et al., 2019). The combinations of glycyrrhizin with entecavir, lamivudine, and other drugs have been reported to yield superior clinical outcomes (Ide et al., 2005; Chen et al., 2020; Minsart et al., 2020; Yu et al., 2020); therefore the potential of combining glycyrrhizin with other drugs must be explored in future studies.

The United States Food and Drug Administration, Council of Europe, and Joint FAO/WHO Expert Committee on Food Additives have allowed the use of liquorice extract and glycyrrhizin in food (Pastorino et al., 2018). Although liquorice is believed to be a healthy natural herb without any serious adverse effects (Kwon et al., 2020), it should be used with caution, particularly in patients with hypertension, considering its side effects. Side effects reported in literature include hypokalaemia (Sontia et al., 2008; Flores-Robles et al., 2013), hypertension (Smedegaard and Svart, 2019), hypertensive encephalopathy (Russo et al., 2000), rhabdomyolysis (Alaygut et al., 2017), and cardiac arrest (Attou et al., 2020). A good correlation has been observed between the content of glycyrrhizin and the incidence of side effects, which indicates that the content of glycyrrhizin is a suitable index to avoid adverse reactions (Nose et al., 2017). However, the upper safe limit of glycyrrhizin remains to be determined. The European Union recommends an upper limit of 100 mg of glycyrrhizin per day (Murphy et al., 2009). Based on *in vivo* results and clinical evidence, Isbrucker et al. recommended that the acceptable daily intake of glycyrrhizin is 0.015–0.229 mg/kg body weight/day (Isbrucker and Burdock, 2006). This range is consistent with the 0.2 mg/kg body weight/day proposed by van Gelderen et al. (2000). Considering the well-known biological functions and side effects of glycyrrhizin, the use of an appropriately controlled dosage of liquorice can lead to its health benefits outweigh its side effects.
